# Marking *Triatoma brasiliensis*, *Triatoma pseudomaculata* and *Rhodnius nasutus* Nymphs with Trace Elements: Element Persistence and Effects of Marking on Insect Mortality

**DOI:** 10.1371/journal.pntd.0004548

**Published:** 2016-03-30

**Authors:** Carolina Valença-Barbosa, Otília Sarquis, Aline Soares Freire, Mariana R. David, Ricardo E. Santelli, Fernando A. Monteiro, Marli M. Lima, Rafael Maciel-de-Freitas

**Affiliations:** 1 Laboratório de Transmissores de Hematozoários, Instituto Oswaldo Cruz, Fiocruz, Rio de Janeiro, Brazil; 2 Laboratório de Ecoepidemiologia da Doença de Chagas, Instituto Oswaldo Cruz, Fiocruz, Rio de Janeiro, Brazil; 3 Laboratório de Desenvolvimento Analítico, Departamento de Química Analítica, Instituto de Química, Universidade Federal do Rio de Janeiro, Rio de Janeiro, Brazil; 4 Laboratório de Epidemiologia e Sistemática Molecular, Instituto Oswaldo Cruz, Fiocruz, Rio de Janeiro, Brazil; University of Perugia, ITALY

## Abstract

**Background:**

Field ecologists often rely on mark-release-recapture (MRR) experiments to estimate population dynamics parameters for a given species. In the case of a medically important taxon, i.e., a disease vector, inferences on species survival and dispersal rates are particularly important as they have the potential to provide insights into disease transmission dynamics in endemic areas. Medical entomologists have traditionally used fluorescent dusts to externally mark the cuticle of insects. However, dust marking is usually restricted to the adult life stage because immature insects lose the mark when they molt.

**Methodology/Principal Findings:**

We evaluated the efficacy of 13 trace elements in marking nymphs of three native Brazilian Chagas disease vectors: *Triatoma brasiliensis*, *Triatoma pseudomaculata*, and *Rhodnius nasutus*. Cr and Cu were detected in over 97% of *T*. *brasiliensis* (34/35 31/31 for Cr and Cu), while Cu and Mn were detected in more than 95% of *T*. *pseudomaculata* (29/29 for Cu and 28/29 for Mn) tested 120 days after marking. Only Mn marked over 90% of *R*. *nasutus* nymphs (38/41). Overall, trace elements had no negative effects on *T*. *pseudomaculata* longevity, but As-marked *T*. *brasiliensis* nymphs (p<0.01), and Cd-marked *R*. *nasutus* nymphs (p<0.01) had significantly shorter lifespan.

**Conclusions/Significance:**

Previous evidence shows that there is little or no genetic differentiation between populations at the microgeographic level, which often precludes indirect estimations of dispersal capability based on genetic markers. In such situations, MRR studies are more suitable as they measure insect movement directly from one site to another, instead of effective migration (i.e. gene flow). The determination of a reliable and persistent marking method is the first step towards the development of meaningful ecological estimates through the application of MRR methodology. Here, we have identified trace elements that can be used for mark and recapture studies of three triatomine species in Brazil.

## Author Summary

Mark, release and recapture (MRR) is one of the most powerful techniques employed by field ecologists, and is used to provide relevant estimates of natural populations such as dispersal, survival rates, and population densities. A reliable, persistent and effective marking method is a *sine qua non* condition to conduct a MRR experiment. Despite their relevance as Chagas disease vectors in the Americas, triatomine bugs have seldom been subjected to MRR studies. So far, estimates on triatomine dispersal have largely relied on molecular/genetic approaches, which are not direct measurements of the distance between the release and recapture point. We evaluated the persistence and efficiency of trace elements, chemicals present in very low quantities in the organism of a targeted species, to mark the nymphs of three Chagas disease vectors of the Brazilian caatinga biome, *Triatoma brasiliensis*, *T*. *pseudomaculata*, and *Rhodnius nasutus*. At least one trace element was shown to be a good marker for each triatomine species: more than 97% of *T*. *brasiliensis* were still marked with Cr and Cu after 120 days; more than 95% of *T*. *pseudomaculata* were marked with Cu and Mn and more than 90% of *R*. *nasutus* were marked with Mn 120 days after marking. Only As-marked *T*. *brasiliensis* and Cd-marked *R*. *nasutus* nymphs had significantly shorter lifespan. We believe these findings show that trace elements are a reliable and persistent marking method for triatomine nymphs and hope this is the first step towards the development of meaningful ecological estimates through the application of the MRR methodology.

## Introduction

Mark-release-recapture (MRR) is an important method used to estimate several ecological parameters of natural populations. For example, species survival, dispersal rates and population density might be inferred based on the collection of previously marked and released individuals. Basically, a subset of a given population is captured, marked, and released. On the subsequent collection effort, another subset is captured and the number of marked (and unmarked) individuals within the same sample is recorded. The number of marked individuals within the second sample should be proportional to the number of marked individuals in the entire population thus, an estimate of population size might be obtained after dividing the number of marked individuals by the proportion of marked individuals in the second sample. Dispersal rates, on the other hand, are simply estimated by measuring the distance between release and recapture points [1].

The success rate of a MRR experiment depends heavily on the reliability of the marking technique and on the effectiveness of the trapping/collection method used. Regarding marking alone, for reliable estimates to be obtained a number of assumptions must be satisfied: [1] marking should not affect the survival rate of marked individuals between sampling events, [2] it should not cause abnormal behavior, which might affect the odds of marked individuals being caught, [3] the mark should be retained during individual lifespan, [4] marked individuals must completely mix in the population before sampling events start and, finally, [5] marked and unmarked individuals must have the same odds of being recaptured [2,3].

Medical entomologists frequently carry out MRR experiments to estimate disease vector population density, survival and dispersal rates [1]. These estimates will certainly help improve vector control policies and practices in endemic regions. Surely, there is no 'universal' marking method that will work flawlessly with all vectors. Vector diversity requires tailor-made techniques to accommodate for any particular idiosyncrasy. For instance, stains and fluorescent powder have been used for almost a century to mark adult mosquitoes [4–7]. However, topical marks such as fluorescent dusts have limited efficacy on immature stages of hemimetabolous insects that go through moulting episodes during their development, as marks are lost with the shedding of the old cuticle.

Despite their relevance as Chagas disease vectors, triatomine bugs have seldom been subjected to MRR studies. Research on triatomine dispersal has largely relied on indirect molecular/genetic approaches based on allozymes, RAPD, microsatellites and mtDNA sequences [8–14]. The active dispersal or flight activity of adult *T*. *infestans*, the primary vector of Chagas disease in Brazil until its eradication in 2006 (a remarkable feat accomplished by the Southern Cone Initiative) [15], has been estimated in several studies [16–19]. However, similar estimates for other triatomine species are still lacking.

Not surprisingly, even less is known about the dispersal capabilities of nymphs, which also have important implications for disease dynamics. We believe that the main reason why so few Chagas vectors MRR studies have hitherto been produced is the absence of simple, reliable, and direct methods to mark triatomine bugs (especially nymphs) in their natural habitat.

Trace elements represent an interesting alternative to the traditional stains and powders to mark insects. A trace element is an element that is needed in very small quantities to allow for the proper working of the organism’s physiology. Therefore, we evaluated the efficiency of trace elements in marking nymphs of three Chagas disease vectors of the Brazilian caatinga biome, *T*. *brasiliensis*, *T*. *pseudomaculata*, and *Rhodnius nasutus*. We also investigated the effects of marking on the lifespan and survival rates of the nymphs. To qualify as a good marker, a trace element must display a long-lasting effect without compromising insect longevity.

## Materials and Methods

### Field collection

Nymphs (N) of *T*. *brasiliensis*, *T*. *pseudomaculata* and *R*. *nasutus* were collected in a Chagas disease endemic area situated in the rural semi-arid region of Jaguaruana, Ceará state, in Northeast Brazil (4°50’14”S 37°46’55”W). Collections were conducted in dwellings, chicken and pigpens and rocks and palm trees on peridomestic areas [20–22]. Collected individuals were brought to the laboratory, identified using taxonomic keys and then used for two main purposes: (1) to determine the natural concentration of chemical trace elements of wild nymphs of each species; and (2) to evaluate the persistence of trace elements on the insects (described below).

### Trace elements on wild triatomine bugs

A first step consisted of assessing trace element concentration in wild triatomine bugs. For that, we collected and determined the concentration of 13 analytes (As, Cd, Cr, Cu, Mn, P, Pb, Rb, S, Sb, Se, Si and Zn) in at least 15 field-collected nymphs per stage from N2 to N5 of *T*. *brasiliensis*, *T*. *pseudomaculata* and *R*. *nasutus*. On a second step, those elements qualified as trace elements and thus suitable candidates to become good markers were later offered in association with sheep blood to bugs to estimate its persistence (see below for further details).

### Persistence of trace element marking on triatomine bugs

We selected 2–4 elements with low concentrations for each species and tested their persistence and potential effects on insect survivorship. We added 0.2 mL of each trace element associated with its chloride form at 0.010 mg L^-1^ to 0.2 mL of defibrinated sheep blood. Each trace element was offered only once to 50 N5 using an artificial feeding apparatus [24]. Subsequent blood feedings were trace elements free and occurred once a month, on anesthetized mice (Fiocruz Ethical Committee LW-24/13). In order to assess whether lifespan was affected by the amount of trace element ingested, insects were subdivided into two categories according to feeding performance efficiency: (1) fully engorged specimens (“Engorged”), or (2) partially engorged (“Unengorged”). Unfed specimens were excluded from the experiment. As controls, 50 N5 individuals of each species received a blood meal free of trace elements on the same membrane feeding apparatus. Persistence of trace element was evaluated on each individual on the day of its death or after a maximum of 120 days, when all live insects were killed by freezing. The same procedure was performed with insects from the control group. All insects that survived the 120 days period were killed as adults.

### Trace element detection

Each triatomine was individually heated to 100°C in 2 mL of 65% nitric acid, as described in Sarquis et al. (2011) [23]. After complete evaporation of the acid, 1 mL of distilled water was added to each sample before analysis by Inductively Coupled Plasma Optical Emission Spectrometry at the wavelengths correspondent to each one of the 13 chemical elements tested. An iCAP 6300 ICP OES model (Thermo Scientific, Cambridge, England) was used in exploratory analyses. After choosing the elements of interest, concentrations were achieved by using an Ultima 2 ICP OES model (Horiba Jobin Yvon, Longjumeau, France). The wavelengths varied from 177.495 nm (As) to 780.023 (Rb)(Table 1).

**Table 1:** Mean ± standard deviation of natural concentration (as mg/L) of 13 chemical elements in wild *Triatoma brasiliensis*, *T*. *pseudomaculata* and *Rhodnius nasutus* nymphs collected in the field site of Jaguaruana, Ceará, Brazil. The appropriate wavelength and the fit of the standard curve for each chemical element tested is indicated. Fifteen specimens were tested per developmental stage, thus comprising a total of 60 individuals per species. When all 15 specimens have the same concentration, no standard deviation was produced.

Trace element	*Triatoma brasiliensis*				*Triatoma pseudomaculata*				*Rhodnius nasutus*				Wavelength	R
	N2	N3	N4	N5	N2	N3	N4	N5	N2	N3	N4	N5		
**As**	0.05	0.05	0.09 ± 0.03	0.20 ± 0.10	0.03	0.03	0.03	0.03	0.03	0.03	0.03	0.03	193,76	0,998
**Cd**	0.03	0.03	0.03	0.03	0.03	0.03	0.03	0.03	0.03	0.03	0.03	0.03	226,51	0,999
**Cr**	0.04 ± 0.02	0.03	0.04 ± 0.01	0.04 ± 0.02	0.03	0.03	0.03	0.03	0.11 ± 0.21	0.11 ± 0.21	0.09 ± 0.08	0.04 ± 0.02	283,56	0,998
**Cu**	0.03	0.04 ± 0.01	0.07 ± 0.05	0.10 ± 0.04	0.10 ± 0.06	0.11 ± 0.03	0.13 ± 0.03	0.14 ± 0.04	0.03	0.03	0.05 ± 0.02	0.13 ± 0.15	324,75	0,996
**Mn**	0.03	0.03	0.03	0.03	0.3	0.03	0.03	0.03	0.03	0.03	0.03 ± 0.01	0.03	257,61	0,997
**P**	12.4 ± 3.3	20.1 ± 9.7	38.1 ± 11.4	89.7 ± 38.1	106.3 ± 62.8	1300 ± 550	4791 ± 1504.2	21647 ± 4171	1.43 ± 0.52	4.7 ± 1.2	17.0 ± 6.5	46 ± 15.8	177,49	0,999
**Pb**	0.04 ± 0.01	0.05 ± 0.02	0.04	0.04	0.03	0.03	0.03	0.03	0.03	0.03	0.03	0.02 ± 0.01	220,35	0,999
**Rb**	0.05 ± 0.01	0.07 ± 0.03	0.14 ± 0.09	0.34 ± 0.16	0.31 ± 0.04	0.35 ± 0.02	0.35 ± 0.02	0.40 ± 0.02	0.07	0.07	0.07	0.11 ± 0.06	780,02	0,991
**S**	10.2 ± 2.8	22.2 ± 12.1	45.8 ± 24.5	113.2 ± 70.4	8.96 ± 3.34	64 ± 30.2	196 ± 83	840 ±156.7	1.44 ± 0.46	4.2 ± 1.0	13.0 ±6.0	34 ± 13.6	180,73	0,995
**Sb**	0.04	0.04	0.04	0.04	0.03	0.03	0.03	0.03	0.03	0.03	0.02 ± 0.03	0.01 ± 0.02	206,83	0,997
**Se**	0.04	0.04	0.04	0.04	0.03	0.03	0.03	0.03	0.03	0.03	0.03	0.03	196,09	0,999
**Si**	0.13 ± 0.01	0.13 ± 0.02	0.13 ± 0.01	0.17 ± 0.03	0.04	0.04	0.04	0.04	0.11 ± 0.04	0.11 ± 0.05	0.17 ± 0.12	0.24 ± 0.14	251,61	0,994
**Zn**	0.37 ± 0.09	0.50 ± 0.13	0.72 ± 0.14	1.5 ± 0.58	0.10 ± 0.07	0.18 ± 0.08	0.29 ± 0.14	0.72 ± 0.15	0.08 ± 0.02	0.10 ± 0.03	0.18 ± 0.05	0.33 ± 0.16	213,85	0,999

Mean absorbance values and standard errors for the controls were used to estimate a confidence interval (α = 0.01). Triatomines from the experimental group whose absorbance values were higher than the superior limit calculated above were considered positive for trace element labeling.

### Effects of trace elements on insect survival

To test the effect of each trace element on insect survival, marked and unmarked (i.e. control) triatomine bugs were monitored daily in their plastic vials with folded filter paper up to 120 days. Alive individuals at the end of experiments were censored for survival analysis. For each species, survival curves were plotted applying the Kaplan-Meier method [25]. In examining the association between trace element marking and triatomine survival, a Cox proportional hazard regression model was adjusted for each species to obtain hazard ratios (HRs) and 95% confidence intervals (CIs). Feeding performance was included in the analysis as a possible confounding factor. The interaction between marking group and feeding performance was excluded from the analysis for *T*. *brasiliensis* due to the absence/low number of unengorged insects per group. When marking significantly affected survival in relation to the control group we tested whether survival of marked triatomine was associated with trace element concentration through a second proportional hazard regression model. When feeding performance affected HRs in marked insects, trace element concentration was compared between engorged and unengorged triatomines with a Kruskall-Walis test (KW). Statistical analyses were performed in R (version 3.2.3) (R Core Team 2015).

## Results

### Trace elements in wild triatomine bugs

As expected, the natural concentration of the 13 chemical elements tested varied among species (Table 1). Most importantly, elements such as As, Cd, Cr, Cu, Mn, Pb, Sb and S presented low and constant concentration whenever tested, indicating they might be classified as trace elements for all the three insect species tested herein.

### Trace element persistence

For *T*. *brasiliensis*, Cr and Cu presented high persistence since more than 97% of insects were still marked until 120 days after the blood meal containing the trace element (Table 2). On the other hand, just a few specimens survived 120 days when marked with As, and the frequency of insects marked with As or Cd was under 75%, indicating that these trace elements were not good markers for *T*. *brasiliensis*.

**Table 2:** Number of *Triatoma brasiliensis*, *Triatoma pseudomaculata* and *Rhodnius nasutus* nymphs blood fed with sheep blood containing trace elements or plain blood, number of insects alive up to the end of survival monitoring and persistence of marking.

	*Triatoma brasiliensis*					*Triatoma pseudomaculata*				*Rhodinius nasutus*		
	As	Cd	Cr	Cu	Control	Cr	Cu	Mn	Control	Cd	Mn	Control
Insects engorged (%)	27 (100)	27 (87.1)	33 (91.6)	26 (83.9)	36 (90.0)	08 (26.7)	13 (44.8)	11 (37.9)	14 (43.8)	09 (21.9)	16 (39.1)	21 (52.5)
Insects unengorged	0	04	03	05	04	22	16	18	18	32	25	19
Insects alive up to the end of monitoring (%)	8 (29.6)	22 (70.9)	35 (97.2)	26 (83.9)	30 (75)	20 (66.7)	24 (82.7)	16 (55.2)	18 (56.2)	5 (12.2)	10 (24.4)	5 (12.5)
Number of marked insects	20 (74.1)	23 (74.2)	35 (97.2)	31 (100)	-	20 (66.7)	29 (100)	28 (96.5)	-	21 (51.2)	38 (92.7)	-

With regard to the three trace elements tested for *T*. *pseudomaculata*, only insects marked with Cu were positive after 120 days, with little mortality observed during the entire four months period. Those marked with Mn presented high efficiency but a more intense mortality, with only 55% of insects surviving the 120 days. Less than 70% of insects marked with Cr were still positive 120 days after marking, suggesting Cr might not be a good marker for *T*. *pseudomaculata* (Table 3).

**Table 3:** Associations between *Triatoma brasiliensis* trace element marking and feeding performance or survival.

Parameter	Coefficient	Hazard Ratio (95% CI)
Marking		
Control		1.0 (reference)
As	1.84	6.30 (2.72–14.60)*
Cd	0.02	1.02 (0.40–2.61)
Cr	-2.31	0.01 (0.01–0.79)
Cu	-0.71	0.49 (0.16–1.50)
Feeding performance		
Unengorged		1.0 (reference)
Engorged	-1.18	0.31 (0.13–0.75)*

* Significant association (p-value < 0.05).

High mortality overall was recorded for *R*. *nasutus*, including those marked with Cd or Mn, but also with those belonging to the control group. Mn was detected in more than 92% of the *R*. *nasutus* 120 days after marking, while Cd effectively marked only 50% of the insects (Table 2).

### Effects of trace element marking on insect survival

**Fig 1:**
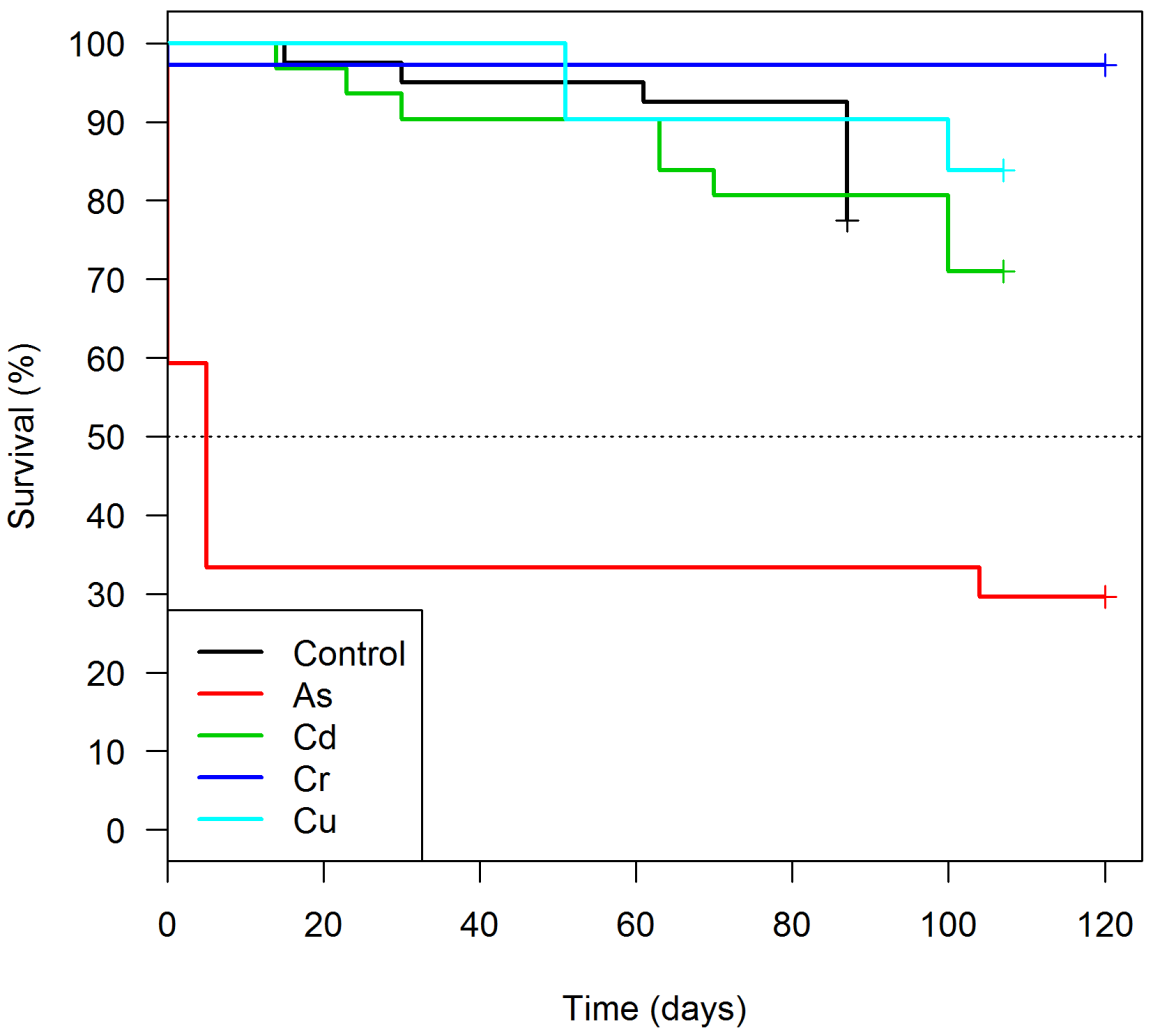
Survival analysis of *Triatoma brasiliensis* fed with sheep blood enriched with one of the following trace elements: As (N = 27), Cd (N = 31), Cr (N = 36), Cu (N = 31) and control group (N = 40).

We found no differences in the survival of *T*. *brasiliensis* marked with Cd (HR: 1.02; p-value = 0.96) or Cu (HR: 0.48; p-value = 0.21) in relation to control. Surprisingly, Cr-marked insects survived more than the control group (HR: 0.09; p-value = 0.03) (Fig 1, Table 3). A high proportion of Cd, Cu and Cr-marked triatomines (71 to 97%) of the insects survived up to 120 days (Table 2).

**Fig 2:**
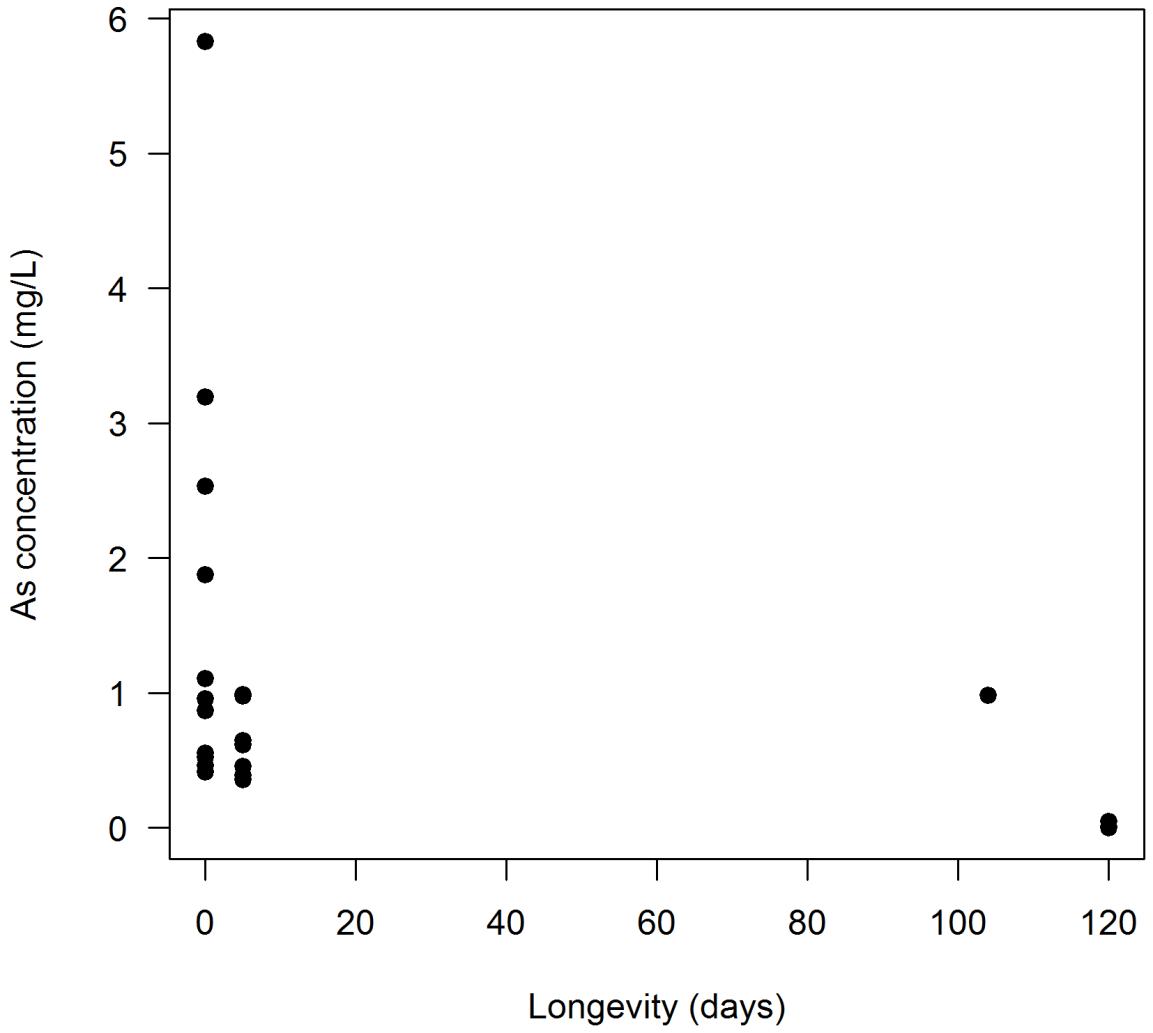
Concentration (mg/L) of arsenic in As-marked *Triatoma brasiliensis* and insect longevity.

On the other hand, As-marked specimens survived significantly less than controls (HR: 6.30; p-value < 0.01), with a high mortality in the first five days after marking, when 19 insects (~70%) died (Fig 1, Table 3). Additionally, survival of As-marked triatomines was negatively associated with As concentration (Coef.:0.49; HR: 1.63; p-value < 0.01) (Fig 2). This outcome is not related to a higher As-blood ingestion, once all insects were engorged (Table 2). Overall, engorged *T*. *brasiliensis* displayed higher survival rates than unengorged specimens (HR: 0.31; p-value < 0.01) (Table 3).

**Fig 3:**
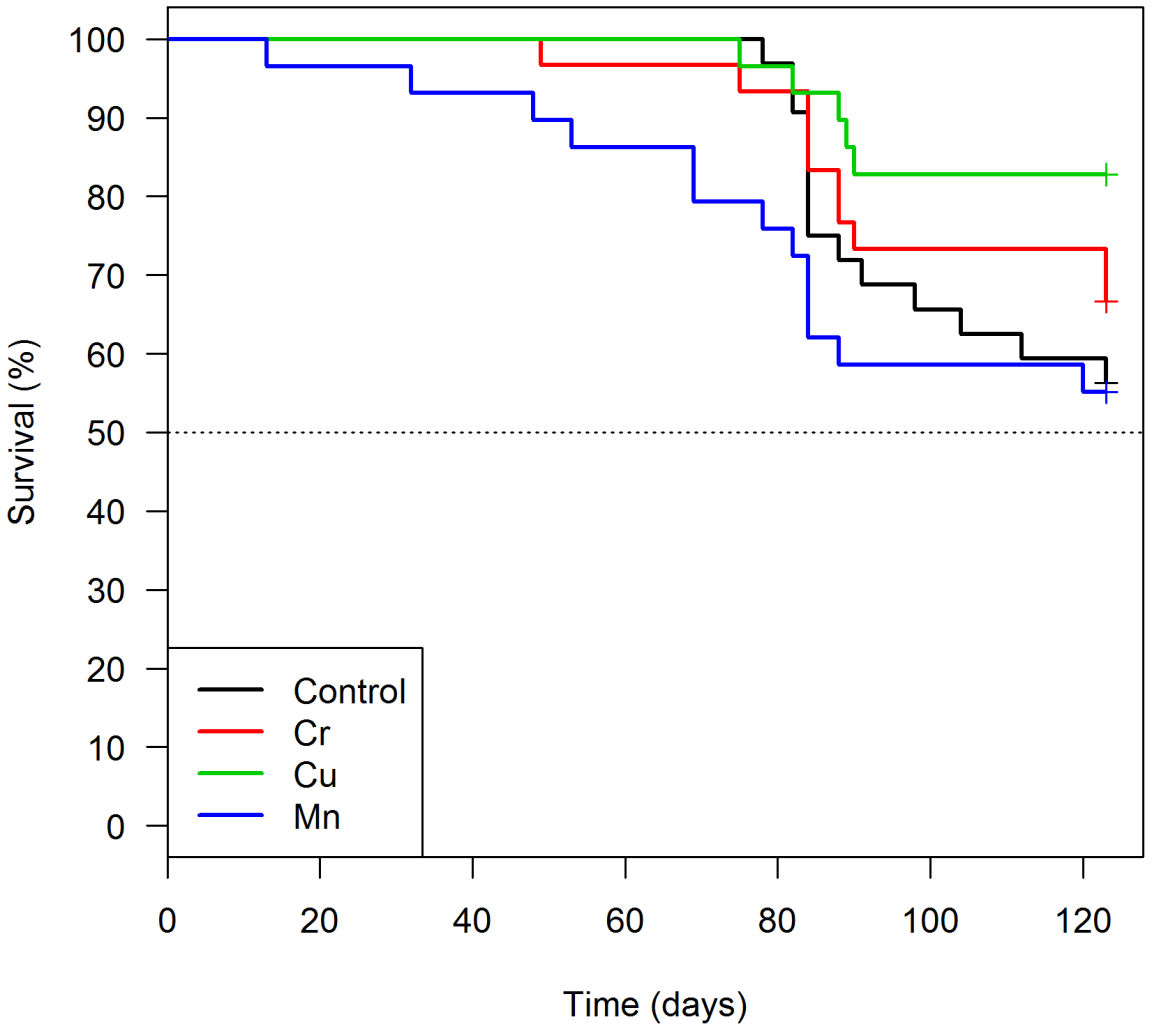
Survival analysis of *Triatoma pseudomaculata* fed with sheep blood enriched with one of the following trace elements: Cr (N = 30), Cu (N = 29), Mn (N = 29) and control group (N = 32).

**Fig 4:**
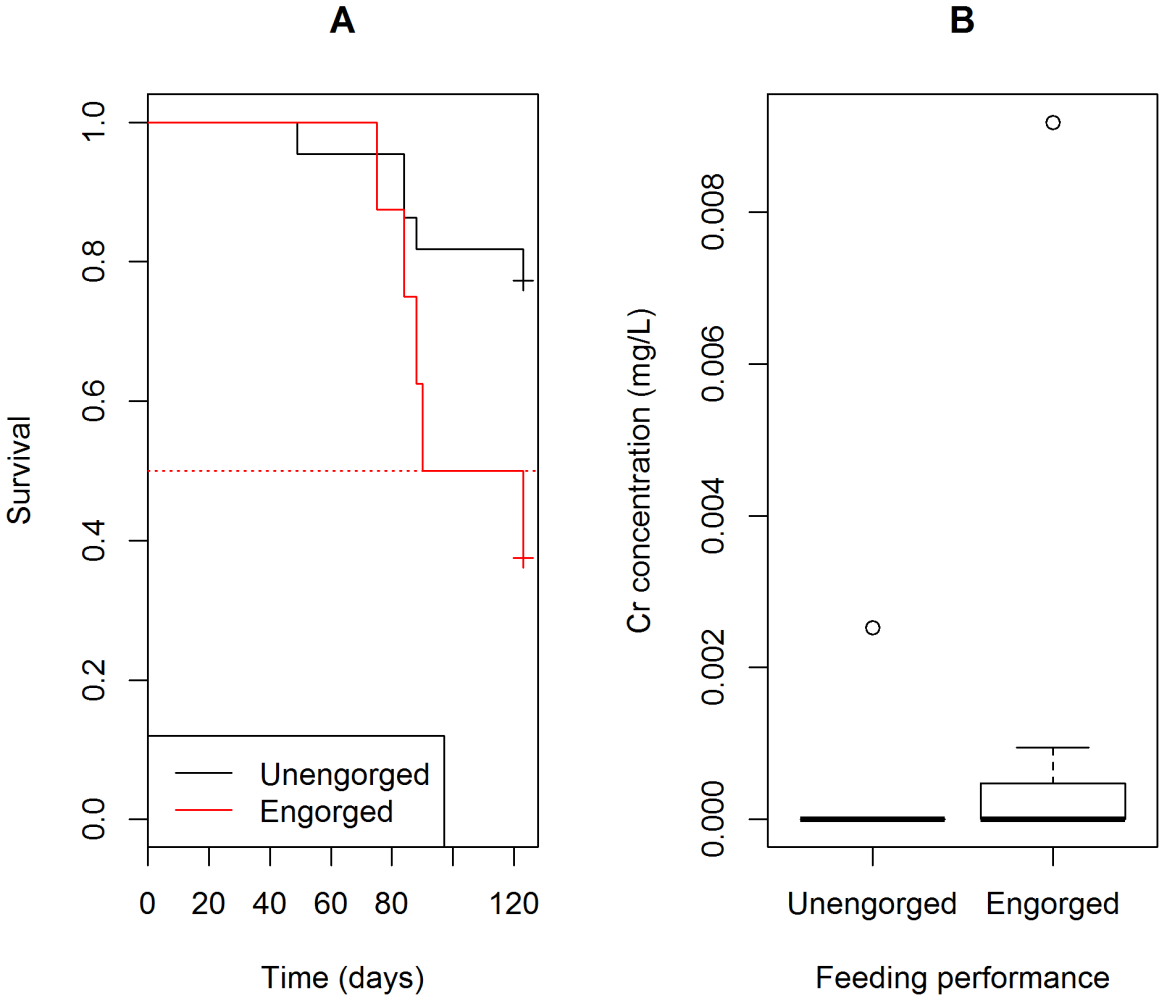
**Survival of Cr-marked *Triatoma pseudomaculata* according to feeding performance (A) and Cr concentration in engorged *versus* unengorged insects (B)**.

For *T*. *pseudomaculata*, marking using Cr (HR: 0.34; p-value = 0.05), Cu (HR: 0.37; p-value = 0.09) or Mn (HR: 0.56; p-value = 0.18) did not significantly alter triatomine survival in comparison to control insects (Fig 3, Table 4). However, feeding performance was associated with mortality in Cr-marked triatomines with a reduced survival in engorged specimens (HR: 7.83; p-value < 0.01) (Table 4). Despite that, feeding performance did not result in distinct Cr concentrations (KW chi-squared = 2.6277, df = 1, p-value = 0.105) (Fig 4).

**Table 4:** Associations between *Triatoma pseudomaculata* trace element marking and feeding performance or survival.

Parameter	Coefficient	HR (95% CI)
Marking		
Control		1.0 (reference)
Cr	-1.084	0.34 (0.11–0.99)
Cu	-0.99	0.37(0.12–1.18)
Mn	0.56	1.76 (0.76–4.07)
Feeding performance		
Unengorged		1.0 (reference)
Engorged	-0.86	0.42 (0.13–1.35)
Interaction		
Cr:unergorged	2.06	7.84 (1.43–42.90
Cu:unergorged	-0.37	0.69 (0.06–8.20)
Mn:unergorged	-1.79	0.17 (0.02–1.75)

**Fig 5:**
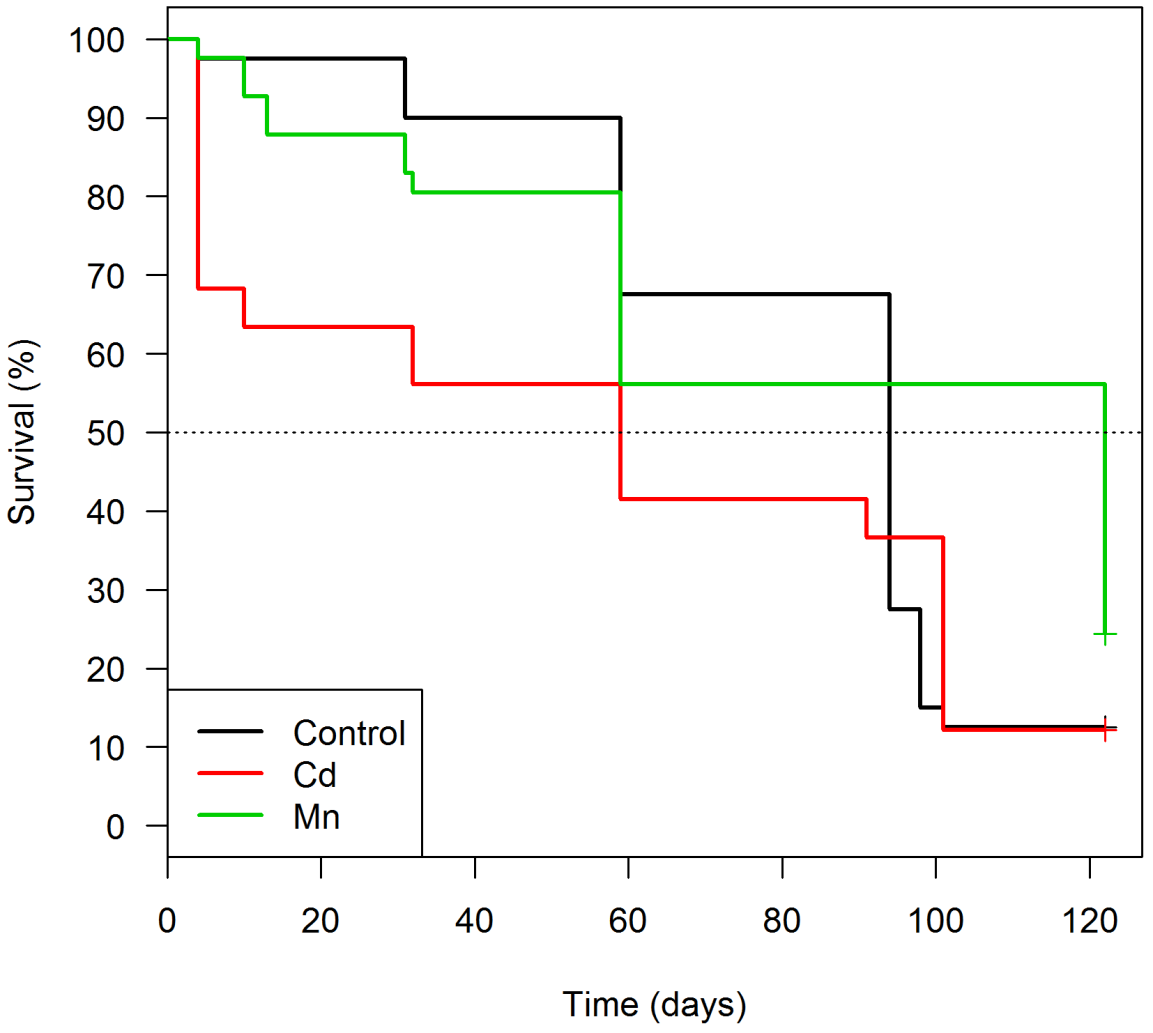
Survival analysis of *Rhodnius nasutus* fed with sheep blood enriched with one of the following trace elements: Cd (N = 41), Mn (N = 41) and control group (N = 40).

**Fig 6:**
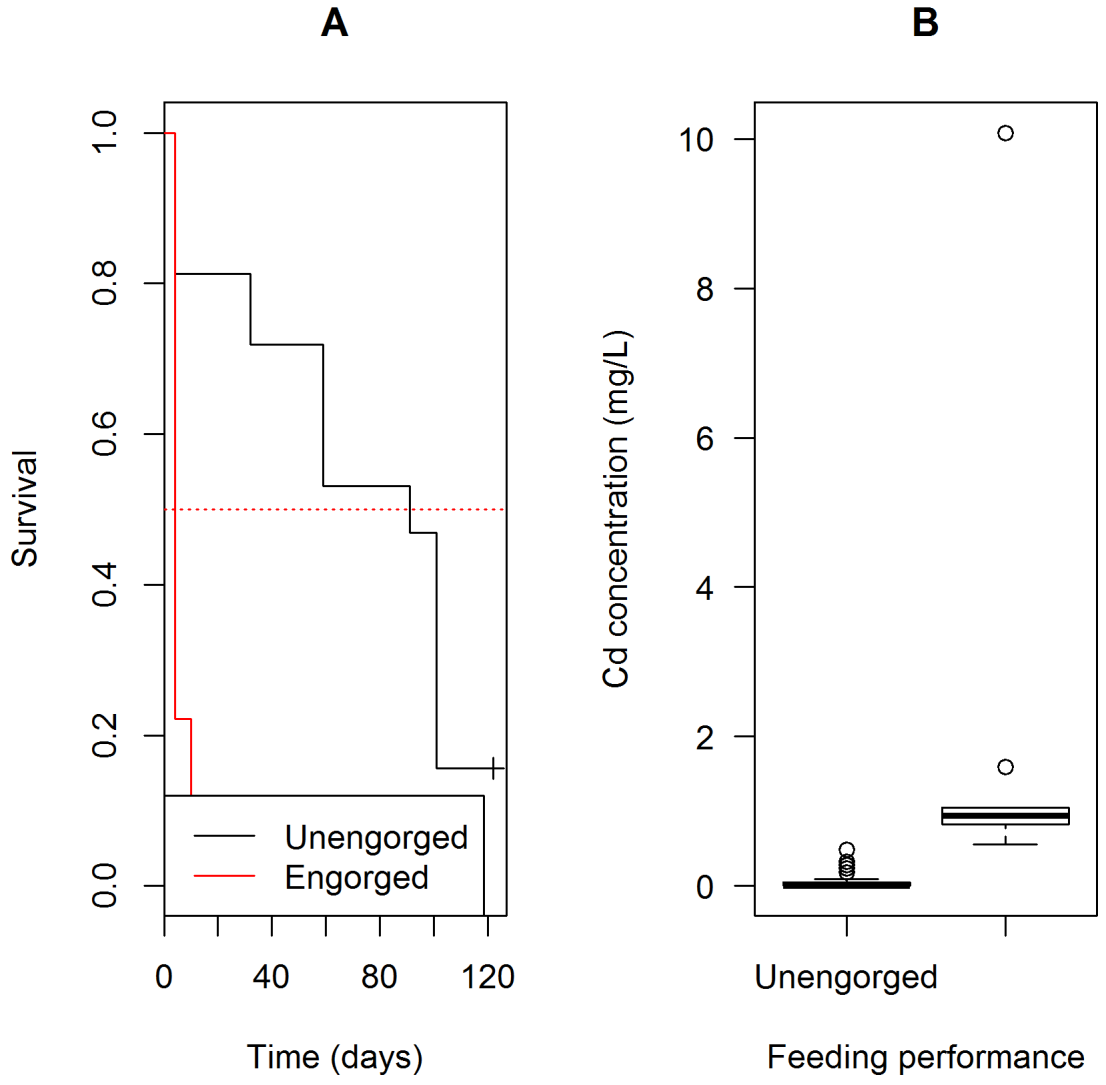
**Survival of Cd-marked *Rhodnius nasutus* according to feeding performance (A) and Cd concentration in engorged *versus* unengorged insects (B)**.

In *R*. *nasutus*, neither Cd (HR: 0.24; p-value = 0.44) or Mn-marking (HR: 0.61; p-value = 0.16) reduced insect survival, although all groups, including the control, showed a high mortality with only 12 to 24% surviving up to 120 days (Fig 5, Tables 2 and 5). Nonetheless, feeding performance was associated with survival in Cd-marked insects with engorged specimens presenting a lower survival (HR: 12.06; p-value < 0.01) (Table 5, Fig 6A). Those insects showed higher Cd body concentrations, which is apparently a consequence of higher blood ingestion during marking assays (Fig 6B).

**Table 5:** Associations between *Rhodnius nasutus* trace element marking and feeding performance or survival.

Parameter	Coefficient	HR (95% CI)
Marking		
Control		1.0 (reference)
Cd	0.24	1.28 (0.68–2.40)
Mn	-0.50	0.61 (0.30–1.23)
Feeding performance		
Unengorged		1.0 (reference)
Engorged	0.66	1.93 (0.98–3.81)
Interaction		
Cd:unergorged	2.49	12.06 (3.66–39.73)*
Mn:unergorged	0.19	1.21 (0.46–3.22)

* Significant association (p-value < 0.05).

## Discussion

Mark-release-recapture (MRR) is an important and powerful method used by field ecologists to estimate biological parameters of the target species on its natural environment. Regarding insects of medical and agricultural relevance, MRR studies have been used to estimate population size, dispersal and survival rates [1,7]. The success of an MRR experiment depends on whether or not the marking method satisfies important requirements such as (i) does not affect survival rates, (ii) does not increase the odds of collecting marked individuals compared to those unmarked, and (iii) is retained during individual lifespan [3]. Herein, we evaluated the use of trace elements to mark three autochthonous Chagas disease vectors of Brazil: *T*. *brasiliensis*, *T*. *pseudomaculata*, and *R*. *nasutus* [26]. Due to the high persistence in the insects’ body, we conclude that Cr and Cu are, amongst the trace elements evaluated, the most suitable markers for *T*. *brasiliensis*, Cu and Mn for *T*. *pseudomaculata*, and Mn for *R*. *nasutus* (Table 2). At the concentration tested, these elements were not toxic and could be detected in a significant number of specimens under laboratory-controlled conditions. In some circumstances, especially in shorter studies, As and Cd may also be used as trace elements because approximately 75% of the individuals were still marked after 120 days.

Animal marking dates back to 200 B.C.E as a tool to help ornithologists to resolve issues concerning bird ownership [27]. Just around 1920, entomologists started using paints, dyes, and stains to determine insect population dynamics. Fluorescent dusts are the most commonly used material for externally marking insets, including disease vectors [1,7]. Dusts are often considered excellent markers because they are inexpensive (thus suitable for mass marking), readily available, environmentally safe, and easily applied and detected under UV light [7]. However, dust marking is usually restricted to the adult life stage because immature insects lose the mark when they molt. For that reason, topical marks such as fluorescent dusts, tags, paints and inks would have limited efficacy for nymphs, but would probably be reliable methods for adults [7]. Some papers have considered the use of immunogenic salivary proteins of *T*. *infestans* as epidemiological markers to detect low-level triatomine infestation in endemic areas [28,29]. Proteins might be used as an internal marking by providing a protein-enriched diet or as an external protein mark topically applied using a medical nebulizer. In both cases, protein detection might be achieved with an ELISA [30]. Focused on presence/absence of disease vectors, immunogenic salivary proteins has the advantage of being low-cost and does not require skilled staff. Trace elements, on the other hand, can provide direct estimates of nymphs’ movement and also quantify dispersal across peridomestic and wild environments, for instance. However, trace-element detection in low-volume samples such as one mL of distilled water requires costly equipment that would probably limit its application for several labs working on Chagas disease. Because some triatomine species will not feed artificially when they come from the field, this step might represent a further challenge. This limitation could be overcome by microinjecting the trace element directly into the vertebrate host and them offer the host to insects [31].

Trace elements in their chloride form have been successfully used to internally mark at least 30 insect families belonging to eight orders, with Rb being the most frequently used marker [1,7]. The majority of reports using trace elements to mark disease vectors have been conducted on dipterans [1, 31–35]. There is one paper published to date that describes the efficiency of Rb and Cr to mark *T*. *brasiliensis* N2-N5 nymphs [23]. Rb marking presented low efficiency, with less than 3% of N3 being marked, but a maximum persistence of 98 days. Cr, on the other hand, marked up to 93% of N5 and persisted up to 119 days on the insects’ body. The survival rate of the Cr-marked group was slightly lower than that observed for the control group. Nonetheless, Cr proved to be useful for the marking of *T*. *brasiliensis* nymphs due to its high positivity and persistence in the insects’ body [23]. Certainly, using trace-elements to mark blood-feeding insects could be further optimized by testing different concentrations of the elements in the blood meal in order to improve vector survival.

Our results showed that the natural concentration of chemical elements varied among the three species tested: *T*. *brasiliensis*, *T*. *pseudomaculata* and *R*. *nasutus*. Most importantly, Rb, which is the trace element most often used to mark insects, can be present in very low concentrations in *R*. *nasutus*, but 3–4 times more concentrated in *T*. *brasiliensis* and *T*. *pseudomaculata*. This variation is somehow surprising if we consider that despite having different habitats, these species occupy the same ecosystem [36,37]. The next step was to select trace elements and evaluate their persistence on the insects’ body and whether they shorten the lifespan of marked individuals.

On a visual basis, *Triatoma brasiliensis* nymphs seemed to blood feed better than *T*. *pseudomaculata* and *R*. *nasutus*. One potential explanation for this observation is that *T*. *brasiliensis* feeds on blood of a wider range of hosts in its natural environment than the other two vector species [38–41]. Nymphs and adults of *T*. *brasiliensis* have been collected with blood contents from a variety of hosts including armadillos, opossum, birds, lizards, and goats. The main food sources of *T*. *pseudomaculata* are birds, rodents and dogs, whereas for *R*. *nasutus*, the main hosts are opossums, and birds [38–42]. Therefore, the use of sheep blood may have favored the species with a more generalist feeding pattern. One limitation of our study is that individuals were not weighed before and after feeding, i.e., we are unaware of the exact concentration of the element ingested. Remarkably, assuming trace elements were homogenized in the sheep blood offered to triatomine bugs, the classes engorged and unengorged indirectly estimated marker ingestion, with the former ingesting more trace elements than the later.

An additional relevant point to determine whether a marking method is effective is to test its potential influence on the mortality of the target species [3]. The mortality analysis conducted with *T*. *brasiliensis* showed that Cr-, Cu- and Cd-marked insects survivorship was comparable to unmarked controls (Fig 1). Furthermore, persistence of Cr and Cu was above 95%, even for insects that survived until 120 days, indicating that these two elements are good markers for this particular species. By considering *T*. *brasiliensis* have an average lifespan of 90–120 days under laboratory conditions, this marking might be present through insect’s life [43]. On the other hand, As-marked insects presented intense mortality in the first five days after the blood meal and also a low marking efficiency (inferior to 75%) (Table 2, Fig 1). These negative effects of As-marking on *T*. *brasiliensis* seemed to be influenced by the concentration of As in the insects’ body, since individuals with higher concentration of As died earlier (Fig 2).

The mortality analysis for *T*. *pseudomaculata* revealed that Cu- and Mn-marking did not increase insect mortality when compared to controls (Fig 3). Marking insects with Cr produced different results in triatomines survival. Cr-marked *T*.*brasiliensis* had an increase in survival, while Cr-marked *T*. *pseudomaculata* had a significant reduction in survival rates over time when compared with their unmarked counterparts. The input of additional amounts of Cr may possibly differentially affect the physiology of *T*. *brasiliensis* and *T*. *pseudomaculata*. With regard to the persistence of trace elements on *T*. *pseudomaculata*, Cu and Mn were the best markers since they were detected in over 95% of the insects studied without affecting longevity. One previous report showed a shorter lifespan for *T*. *pseudomaculata*, since adults lived for 221 ± 21 days (mean ± SD) [44]. For *R*. *nasutus*, adult males and females have an average lifespan of 145 and 114 days, respectively [45], Cd and Mn did not affect insect survival, while the later was indicated as the best marker since it was detectable in more than 95% of the individuals tested. Interestingly, the individuals that died prematurely had higher Cd concentrations than those that survived up to 120 days, indicating that *R*. *nasutus* may be intolerant to high concentrations of Cd.

In order to be elected as a good marker, the trace element tested must persist in the insect for a long period of time, say 120 days, without compromising survival rate. In conclusion, we showed which trace elements were suitable markers for *T*. *brasiliensis*, *T*. *pseudomaculata* and *R*. *nasutus* nymphs. We hope the trace elements described here are useful for those interested in the estimation of vector population parameters such as insect dispersal, survival rates, habitat selection and host-seeking behavior under natural conditions.

Although the genetic structure and gene flow among triatomine populations has been effectively determined in macro-geographical scales, as exemplified in the Introduction section, molecular markers seem to not be as useful for small-scale dispersion studies [46]. When the geographic distance between populations is minute, most likely there will be no population substructure (i.e. panmixia will predominate), which will inevitably prevent the estimation of traditional gene flow parameters, as all individuals will likely be part of the same gene pool [13]. It should also be kept in mind that whereas MRR studies measure insect movement directly, most indirect genetic methods estimate “effective migration” instead (i.e. migration followed by reproduction; [47].
